# Fasting glucose and risk of colorectal cancer in the Korean Multi-center Cancer Cohort

**DOI:** 10.1371/journal.pone.0188465

**Published:** 2017-11-21

**Authors:** Hyeree Park, Sooyoung Cho, Hyeongtaek Woo, Sue K. Park, Hai-Rim Shin, Soung-Hoon Chang, Keun-Young Yoo, Aesun Shin

**Affiliations:** 1 Seoul National University College of Medicine, Seoul, Republic of Korea; 2 Department of Preventive Medicine, Seoul National University College of Medicine, Seoul, Republic of Korea; 3 Department of Biomedical Science, Seoul National University College of Medicine, Seoul, Republic of Korea; 4 Cancer Research Center, Seoul National University, Seoul, Republic of Korea; 5 World Health Organization Regional Office for the Western Pacific, Manila, Philippines; 6 Department of Preventive Medicine, Konkuk University School of Medicine, Chungju, Republic of Korea; Shanghai Diabetes Institute, CHINA

## Abstract

Previous cohort studies have demonstrated a positive association between diabetes mellitus (DM) and colorectal cancer (CRC). However, there are few comparisons between DM groups categorized by fasting glucose level. This study examined associations between diabetes as defined by fasting glucose level and self-reported history of DM and CRC risk among Korean adults. Data from the Korean Multi-center Cancer Cohort between 1993 and 2005 were analyzed. The study population comprised 14,570 participants aged 20 years or older. Participants were followed until December 31, 2012 (median follow-up: 11.9 years). Among participants with high fasting glucose (≥126mg/dL), the risk of developing CRC was significantly higher (HR: 1.51 [1.02–2.25]) than among participants with low fasting glucose (<126mg/dL). Risk was not significantly higher among participants with self-reported history of DM (HR: 1.34 [0.78–2.31]). When both fasting glucose and history of DM were considered together, the risk of CRC among participants with both high fasting glucose and history of DM was 54% (HR: 1.54 [0.97–2.43]), and the risk of CRC among participants with high fasting glucose and no history of DM was 50% (HR: 1.50 [0.73–3.05]). When the first 5 years of follow-up were excluded, among participants with high fasting glucose, the risk of developing CRC was significantly higher (HR: 1.61 [1.02–2.56]) than among participants with low fasting glucose. Risk of CRC was also significantly higher among participants with high fasting glucose and no history of DM (HR: 1.69 [1.01–2.84]). High fasting glucose and self-reported history of DM were associated with increased risk of CRC in this Korean population.

## Introduction

According to the Korea Central Cancer Registry, 225,343 individuals (111,599 men; 113,744 women) were diagnosed cancer in 2015, and the lifetime risk of developing cancer was 38.3% for men and 35.0% for women. [1] Colorectal cancer is the second most common cancer among men, the third most common cancer among women, and the third most common cancer overall in Korea. The age-standardized incidence rate per 100,000 population for colorectal cancer in 2013 was 46.9 for men and 25.5 for women. The age-standardized incidence rate for both sexes steadily increased until 2011; the rate started to decline in 2012, especially among men. [1] Despite this decrease, the incidence rate for colorectal cancer is still higher compared with other types of cancer. Studying the correlation of various environmental risk factors and the prevalence of colorectal cancer is crucial.

Incidence of diabetes mellitus is increasing worldwide. [2] In Korea, mortality due to diabetes mellitus and its complications has also been increasing. [3] According to the Korean Statistics Office, the age-standardized mortality rate for diabetes mellitus was 12.8 per 100,000 population in 2015, which was lower than the previous year. [4] Among individuals aged 30 years or older, the age-standardized prevalence of diabetes mellitus was close to 9% for the decade between 2000 and 2010 and increased to 10.2% in 2014. Among all Koreans aged 30 years or older, the prevalence of diabetes mellitus was 9.5% in 2015; among men prevalence was 11.0% and among women it was 8.0%. [5] However, awareness of diabetes mellitus among Koreans aged 30 years or older, i.e., the proportion of people with diabetes mellitus who were aware of their condition was about 70% between 2008 and 2010, and had not improved from 2005. [6] Awareness was shown to be higher among women than among men, and among older patients. The treatment rate (the proportion of people treated with glucose-lowering medications or insulin injections) was about 60% and was also higher among women and older patients.

Some studies have reported higher risk of colorectal cancer among patients with diabetes mellitus compared with individuals who do not have diabetes mellitus. Common known risk factors for both diabetes mellitus and colorectal cancer include Western diet, obesity, smoking, and low levels of physical activity. [7] Four meta-analysis studies on the association of diabetes mellitus and the risk of colorectal cancer have included around 33 cohort studies. [7–10] When we compared the risk ratio of colorectal cancer according to sex in the 17 cohort studies in which the study population included both sexes, risk for colorectal cancer was higher among men than among women in 12 studies; 5 studies showed otherwise. [9, 10] In a previous study conducted in Korea on the association of fasting serum glucose level and risk of cancer, women had higher risk (relative risk [RR]: 1.17 [0.98–1.40]) than men (RR 1.11 [1.00–1.24]). Among men with high fasting serum glucose (FSG) (≥140mg/dL), the mortality ratio due to colorectal cancer was 1.31 [1.03–1.67] and was statistically significant, whereas the same association for women was not statistically significant. [11]

In this study we assessed the association between diabetes mellitus and colorectal cancer risk in the Korean population. The objective of the study was to investigate and compare colorectal cancer risk among participants of the Korean Multi-center Cancer Cohort study with diabetes mellitus in groups defined by fasting glucose measurements and self-reported history of diabetes mellitus.

## Materials and methods

### Study population

The Korean Multi-center Cancer Cohort is a population-based prospective cohort study designed to investigate the relationship between exposures to environmental factors, lifestyle factors, and the risk of cancer in Korea. [12] A total of 20,636 participants (8,235 men and 12,401 women) were recruited from six geographic areas of Korea from 1993 to 2005. For these analyses, we excluded participants who had no information on age at cohort recruitment (1,182 men and 1,792 women), whose fasting glucose level information was incomplete or who had no information about history of diabetes mellitus (1,007 men and 1,575 women), who were aged 20 years or younger (156 men and 334 women), who had a prior diagnosis of colorectal cancer (6 men and 13 women), or who were censored within one month from the baseline survey (1 man). After these exclusions, 14,570 participants (5,883 men and 8,687 women) were included in the final analysis. This study was approved by the Institutional Review Board (IRB) at Seoul National University Hospital (IRB number: 1407-097-597).

### Data collection

Study participants answered questions about age, education level, cigarette smoking and alcohol drinking habits, regular exercise status and history of cancer. The structured study questionnaire was administered by trained interviewers. [13] Body mass index (BMI) was calculated by dividing weight by height squared and categorized as <18.5, 18.5–22, 23–24, and ≥25. Height and weight information was directly measured using standard methods at the time of the physical examination. [12] Participants’ level of physical activity was determined by asking questions about how long they engaged in various moderate physical activities (i.e., walking, playing golf, bowling, riding a bicycle on a flat road, sports, dancing, cleaning, etc.). Participants were also asked whether a physician had ever told them they had diabetes mellitus or a colorectal polyp. Following a standard protocol, participants fasted for at least 6 hours before blood collection. After sampling whole blood in serum-separating tubes, centrifugation of the sample followed in order to separate the serum. The hexokinase method was used for in vitro quantitative measurement of glucose concentrations.

### Statistical analysis

Cohort participants were followed for cancer occurrence by using the Korea Central Cancer Registry database and for deaths up to December 31, 2012 by linking to the death certificate database of the Korean Statistics Office. Colorectal cancer cases were defined using ICD-10 codes C18 (malignant carcinoid tumors of the colon), C19 (malignant neoplasm rectosigmoid junction), and C20 (malignant neoplasm of rectum). [14]

The follow-up period for each participant was defined as the duration from recruitment to the end of follow-up. In the case of colorectal cancer cases, the end of follow-up was defined as the diagnosis date. For participants who were not diagnosed with colorectal cancer, the end of follow-up was defined as the date of death or December 31, 2012. The population as of the middle of the year 2000 was used as the standard population in the direct standardization.

Fasting glucose level and history of diabetes mellitus were both used as criteria for defining diabetes mellitus cases. Participants who had reported being previously diagnosed with diabetes mellitus by a doctor were defined as a diabetes mellitus case. Participants were also categorized according to their fasting glucose level as follows: normal group (fasting glucose < 126mg/dL) or diabetic group (fasting glucose ≥ 126mg/dL) by using the World Health Organization and American Diabetes Association criteria. [15, 16] Participants were further categorized using both definitions as follows: normal (fasting glucose < 126mg/dL and no history of diabetes mellitus), fasting glucose < 126mg/dL and a history of diabetes mellitus, fasting glucose ≥126mg/dL and a history of diabetes mellitus, and undiagnosed (fasting glucose ≥126mg/dL and no history of diabetes mellitus).

We used the Pearson chi-square test to assess the association between fasting glucose level and demographic variables. A Cox proportional hazards model was used to estimate the hazard ratios (HRs) and 95% confidence intervals (CIs) of colorectal cancer according to the fasting glucose level. The time scale used in the Cox model was age during follow-up. The potential confounding variables considered were sex, education level, physical activity level, BMI, cigarette smoking, alcohol consumption, and geographic area. After conducting a likelihood ratio test, adjustments were made only for sex and geographic area. All statistical analyses were performed by using SAS software version 9.4 (SAS Institute Inc., Cary, North Carolina, United States).

## Results

Table 1 shows the general characteristics of the participants according to fasting glucose level. The average age of study participants was 54.3 years for men (standard deviation: 14.2) and 55.2 years for women (standard deviation: 13.0). A total of 728 participants (279 men, 449 women) reported a history of diabetes mellitus, and 49.5% of men and 52.8% of women with prior diagnosis of diabetes mellitus had higher fasting glucose level. During a median of 11.9 years of follow-up and 181,655 person-years of observation, there were 189 colorectal cancer cases (97 men and 92 women). A total of 35 participants (22 men and 13 women) reported a history of colorectal polyps. More than half of participants were aged 50 to 69 years at baseline, and the majority of participants with a prior diagnosis of diabetes mellitus were older than age 60 years. Participants with history of diabetes mellitus had higher BMI than their counterparts with no diabetes mellitus history. The proportion of current drinkers and current smokers was higher in normal glucose-level group compared to the diabetes mellitus group.

**Table 1:** General characteristics of the study participants according to history of diabetes mellitus (DM) and fasting glucose levels, Korean Multi-center Cancer Cohort, 1993–2005.

Variable	No history of DM & glucose < 126 mg/dL	History of DM & glucose < 126 mg/dL	History of DM & glucose ≥ 126 mg/dL	No history of DM & glucose ≥ 126 mg/dL	Total
N (%)	12,911 (88.6)	353 (2.4)	375 (2.6)	931 (6.39)	14,570
Sex					
Men	5,193 (40.2)	141 (39.9)	138 (36.8)	411 (44.2)	5,883 (40.4)
Women	7,718 (59.8)	212 (60.1)	237 (63.2)	520 (55.9)	8,687 (59.6)
Colorectal cancer cases	154	6	8	21	189 (1.3)
History of colorectal polyps					
Yes	32 (0.2)	2 (0.6)	0	1 (0.1)	35 (0.2)
No	10,830 (83.9)	301 (85.3)	325 (86.7)	802 (86.1)	12,258 (84.1)
Unknown	28 (0.2)	2 (0.6)	0	2 (0.2)	32 (0.2)
Missing	2,021 (15.7)	48 (13.6)	50 (13.3)	126 (13.5)	2,245 (15.4)
Age at baseline (years)					
20–29	587 (4.6)	4 (1.1)	0 (0.0)	13 (1.4)	604 (4.1)
30–39	1,673 (13.0)	14 (4.0)	5 (1.3)	66 (7.1)	1,758 (12.1)
40–49	2,383 (18.5)	30 (8.5)	43 (11.5)	145 (15.6)	2,601 (17.9)
50–59	3,225 (25.0)	94 (26.6)	107 (28.5)	258 (27.7)	3,684 (25.3)
60–69	3,577 (27.7)	150 (42.5)	163 (43.5)	299 (32.1)	4,189 (28.8)
70–79	1,322 (10.2)	53 (15.0)	52 (13.9)	127 (13.6)	1,554 (10.7)
80–89	138 (1.1)	8 (2.3)	5 (1.3)	23 (2.5)	174 (1.2)
≥ 90	6 (0.1)	0	0	0	6 (0.0)
Education level (years)					
No formal education	2,596 (20.1)	99 (28.1)	95 (25.3)	263 (28.3)	3,053 (21.0)
1–12	9,848 (76.3)	239 (67.7)	267 (71.2)	642 (69.0)	10,996 (75.5)
≥ 13	417 (3.2)	13 (3.7)	13 (3.5)	23 (2.5)	466 (3.2)
Missing	50 (0.4)	2 (0.6)		3 (0.3)	55 (0.4)
Total time spent participating in moderate physical activity (hour/week)					
Never	2,625 (20.3)	56 (15.9)	86 (22.9)	210 (22.6)	2,977 (20.4)
0.5–7	4,513 (35.0)	147 (41.6)	141 (37.6)	321 (34.5)	5,122 (35.2)
7–21	2,319 (18.0)	59 (16.7)	69 (18.4)	164 (17.6)	2,611 (17.9)
> 21	1,251 (9.7)	43 (12.2)	29 (7.7)	101 (10.9)	1,424 (9.8)
Unknown	2,131 (16.5)	47 (13.3)	49 (13.1)	129 (13.9)	2,356 (16.2)
Missing	72 (0.6)	1 (0.3)	1 (0.3)	6 (0.6)	80 (0.5)
Body mass index (kg/m^2^)					
< 18.5	497 (3.9)	4 (1.1)	7 (1.9)	40 (4.3)	548 (3.8)
18.5–22	5,032 (39.0)	89 (25.2)	94 (25.1)	293 (31.5)	5,508 (37.8)
23–24	2,919 (22.6)	105 (29.8)	97 (25.9)	189 (20.3)	3,310 (22.7)
≥ 25	3,830 (29.7)	147 (41.6)	158 (42.1)	369 (39.6)	4,504 (30.9)
Missing	633 (4.9)	8 (2.3)	19 (5.1)	40 (4.3)	700 (4.8)
Alcohol consumption history					
Never drinker	7,113 (55.1)	195 (55.2)	223 (59.5)	520 (55.9)	8,051 (55.3)
Former drinker	706 (5.5)	44 (12.5)	25 (6.7)	55 (5.9)	830 (5.7)
Current drinker	4,979 (38.6)	112 (31.7)	125 (33.3)	352 (37.8)	5,568 (38.2)
Missing	113 (0.9)	2 (0.6)	2 (0.5)	4 (0.4)	121 (0.8)
Cigarette smoking history					
Never smoker	7,998 (62.0)	221 (62.6)	249 (66.4)	539 (57.9)	9,007 (61.8)
Former smoker	1,422 (11.0)	55 (15.6)	50 (13.3)	103 (11.1)	1,630 (11.2)
Current smoker	3,410 (26.4)	76 (21.5)	76 (20.3)	283 (30.4)	3,845 (26.4)
Missing	81 (0.6)	1 (0.3)	0	6 (0.6)	88 (0.60)

The HRs and 95% CIs for colorectal cancer according to fasting glucose level are shown in Table 2. Among participants with high fasting glucose (≥126mg/dL), the risk of developing colorectal cancer was higher (HR: 1.51 [1.02–2.25]) compared with participants with low fasting glucose (<126mg/dL). Risk was also higher among participants with a history of diabetes mellitus (HR: 1.34 [0.78–2.31]), although the difference was not statistically significant. Female participants with a self-reported diagnosis of diabetes by a doctor had significantly increased risk (HR: 2.07 [1.07–4.00]). When both fasting glucose and history of diabetes mellitus were considered, the risk of colorectal cancer was non-significantly elevated among participants with high fasting glucose and no history of diabetes mellitus (HR: 1.54 [0.97–2.43]). Participants with high fasting glucose and a history of diabetes mellitus had a non-significantly elevated risk of colorectal cancer (HR: 1.50 [0.73–3.05]).

**Table 2:** Hazard ratios (HRs) and 95% confidence intervals (CIs) for colorectal cancer according to fasting glucose level and history of diabetes mellitus (DM) in the Korean Multi-center Cancer Cohort, 1993–2005.

	Both sexes*				Men**				Women**			
	Number	CRC cases (n)	Person-years	HR* (95% CI)	Number	CRC cases (n)	Person-years	HR** (95% CI)	Number	CRC cases (n)	Person-years	HR** (95% CI)
Fasting glucose level												
< 126mg/dL	13,264	160	164,421	1.00 (Ref.)	5,334	81	63,775	1.00 (Ref.)	7,930	79	100,645	1.00 (Ref.)
≥ 126mg/dL	1,306	29	17,234	1.51 (1.02–2.25)	549	16	7,117	1.60 (0.93–2.74)	757	13	10,117	1.40 (0.78–2.54)
Per 10 mg/dL increase				1.02 (0.99–1.05)				1.01 (0.97–1.06)				1.03 (0.99–1.07)
Self-reported history of diabetes mellitus												
No	13,842	175	173,385	1.00 (Ref.)	5,604	93	67,661	1.00 (Ref.)	8,238	82	105,723	1.00 (Ref.)
Yes	728	14	8,270	1.34 (0.78–2.31)	279	4	3,231	0.72 (0.26–1.95)	449	10	5,039	2.07 (1.07–4.00)
Fasting glucose level and self-reported history of diabetes mellitus												
No history of DM & Glucose < 126mg/dL	12,911	154	160,581	1.00 (Ref.)	5,193	79	62,220	1.00 (Ref.)	7,718	75	98,361	1.00 (Ref.)
History of DM & Glucose < 126mg/dL	353	6	3,840	1.28 (0.56–2.90)	141	2	1,555	0.79 (0.19–3.24)	212	4	2,284	1.87 (0.68–5.14)
History of DM & Glucose ≥ 126mg/dL	375	8	4,431	1.50 (0.73–3.05)	138	2	1,676	0.75 (0.18–3.05)	237	6	2,755	2.25 (0.98–5.18)
No history of DM and Glucose ≥ 126mg/dL	931	21	12,803	1.54 (0.97–2.43)	411	14	5,441	1.90 (1.07–3.37)	520	7	7,362	1.10 (0.50–2.39)

*Adjusted for sex and area.

**Adjusted for area.

Since few colorectal cancer cases occurred during the early period of follow-up, a sensitivity analysis was conducted by excluding the first 2 (S1 Table), 5, and 10 (S2 Table) years of follow-up. The HRs and 95% CIs for colorectal cancer according to fasting glucose level when the first 5 years of follow-up was excluded are shown in Table 3. Among participants with high fasting glucose (≥126mg/dL), the risk of developing colorectal cancer was significantly higher (HR: 1.61 [1.02–2.56]) compared with participants with low fasting glucose (<126mg/dL). When fasting glucose and history of diabetes mellitus were considered together, the risk of colorectal cancer was significantly higher among participants with high fasting glucose and no history of diabetes mellitus (HR: 1.69 [1.01–2.84]).

**Table 3:** Hazard ratios (HRs) and 95% confidence intervals (CIs) for colorectal cancer according to fasting glucose level and history of diabetes mellitus (DM) after excluding the first 5 years of follow-up in the Korean Multi-center Cancer Cohort, 1993–2005.

	Both sexes*				Men**				Women**			
	Number	CRC cases (n)	Person-years	HR* (95% CI)	Number	CRC cases (n)	Person-years	HR** (95% CI)	Number	CRC cases (n)	Person-years	HR** (95% CI)
Fasting glucose level	14,311	132			5,703	67			8,608	65		
< 126mg/dL	13,031	110	98,602	1.00 (Ref.)	5,166	54	37,475	1.00 (Ref.)	7,865	56	61,128	1.00 (Ref.)
≥ 126mg/dL	1,280	22	10,764	1.61 (1.02–2.56)	537	13	4,397	1.81 (0.98–3.34)	743	9	6,367	1.39 (0.68–2.83)
Per 10 mg/dL increase				1.03 (1.00–1.06)				1.02 (0.97–1.07)				1.04 (1.00–1.08)
Self-reported history of diabetes mellitus												
No	13,604	123	104,693	1.00 (Ref.)	5,433	64	40,018	1.00 (Ref.)	8,171	59	64,674	1.00 (Ref.)
Yes	707	9	4,673	1.37 (0.70–2.71)	270	3	1,853	0.84 (0.26–2.70)	437	6	2,820	2.06 (0.89–4.81)
Fasting glucose and self-reported history of diabetes mellitus												
No history of DM & Glucose < 126mg/dL	12,689	106	96,510	1.00 (Ref.)	5,031	52	36,615	1.00 (Ref.)	7,658	54	59,895	1.00 (Ref.)
History of DM & Glucose < 126mg/dL	342	4	2,092	1.47 (0.54–3.99)	135	2	860	1.37 (0.33–5.68)	207	2	1,233	1.66 (0.40–6.83)
History of DM & Glucose ≥ 126mg/dL	365	5	2,581	1.45 (0.59–3.57)	135	1	994	0.58 (0.08–4.22)	230	4	1,587	2.37 (0.86–6.59)
No history of DM and Glucose ≥ 126mg/dL	915	17	8,183	1.69 (1.01–2.84)	402	12	3,403	0.01 (1.18–4.21)	513	5	4,779	1.07 (0.42–2.68)

*Adjusted for sex and area.

**Adjusted for area.

When glucose level was considered as a continuous variable, colorectal cancer risk increased incrementally according to each 10 mg/dL change in fasting glucose level (Tables 2 and 3). When all participants were included in the analysis, the risk of colorectal cancer rose by 2% per 10 mg/dL change in fasting glucose level (Table 2). When the first 5 years of follow-up were excluded, the risk of developing colorectal cancer rose by 3% per 10 mg/dL change in fasting glucose level (Table 3). No statistically significant elevation in colorectal cancer risk was observed per 10 mg/dL increment among women (HR: 1.03 [0.99–1.07]) or men (HR: 1.01 [0.97–1.06]) when the sexes were considered separately. After exclusion of 5 years of follow-up, a significant increase in colorectal cancer risk was observed among women (HR: 1.04 [1.00–1.08]).

## Discussion

This study found that fasting glucose level and self-reported history of diabetes mellitus were each related to increased risk for colorectal cancer. When both fasting glucose and history of diabetes mellitus were considered together as indicators of diabetes, the risk for colorectal cancer also increased.

After direct standardization of the population, incidence of colorectal cancer among participants aged 20 years or older per 100,000 population was 51.5 for men, and 37.7 for women. This was lower than the indirectly age-standardized incidence rate of colorectal cancer in 2012 among Koreans aged 20 years or older (64.0 men and 44.5 women), whereas it was higher than the age-standardized incidence rate in 1999 (33.2 men, 26.6 women). Considering that colorectal cancer incidence had rapidly increased between 1999 and 2012 in Korea, the incidence rate among Korean Multi-center Cancer Cohort participants is comparable to the colorectal cancer incidence in Korean population. [17]

In our study, when diabetes mellitus was defined as the proportion of people whose fasting glucose level was ≥126mg/dL or as the proportion of people who self-reported diagnosis of diabetes by a doctor, prevalence was 12.6%. According to the Korean National Health and Nutrition Examination Survey I (KHANES I) report in 1998, the prevalence of diabetes mellitus in adults aged 20 years or older was 9.3% (men, 10.5%; women, 8.2%). [18] The age standardized prevalence was 21.0% among all adults aged 50 to 59 years, 18.7% among those aged 60 to 69 years, and 15.8% among those aged 70 years or older. [18] Considering that more than half of our study’s participants were over age 50 years, the prevalence of diabetes mellitus that we found is higher than that reported by the 1998 study. Prevalence appeared to increase with age and was as high as 20% among Koreans aged 50 or older with diabetes mellitus among both sexes. This prevalence is similar to that reported by KHANES in 2005. [19]

The awareness rate in our study—the proportion of people whose fasting glucose level was ≥126mg/dL or who reported a diagnosis of diabetes mellitus—was 39.3% in 1995 when the baseline survey was first conducted. The awareness rate according to KHANES was 40.4% in 1998, which increased to 43% in 2001 and then to 66.5% in 2005. [18] Probable explanations for this phenomenon may be that 20 years ago awareness of diabetes mellitus in the general population was uncommon and the health check-up service that is now available to every citizen did not exist.

People who were previously diagnosed as having diabetes mellitus but whose fasting glucose levels were lower than 126mg/dL comprised 18.9% of diabetic patients in our study population. Since Hb1Ac was not measured during the baseline survey (the time of cohort recruitment), no control rate could be calculated. In 2013, the control rate of diabetes mellitus in people aged 50 years or older was about 20%. [6]

Risk for colorectal cancer was 57% higher among participants with high fasting glucose levels and a history of diabetes mellitus, compared with participants in the normal fasting glucose level group and no history of diabetes mellitus. According to the National Health Insurance Service cohort study in 2005, risk for colorectal cancer among participants with diabetes mellitus (FSG >126mg/dL) compared with the control group (FSG <90mg/dL) was 1.13 (95% CI: 1.03–1.23; adjusted for age, cigarette smoking, and alcohol consumption). [11] The Cardiovascular Health Study cohort reported that the highest FSG level group (>140 mg/dL or diagnosed with diabetes mellitus) had an 80% higher risk (95% CI: 1.0–3.1) of developing colorectal cancer compared with participants in the lowest FSG level group, which is higher than our result (adjusted for age, sex, and physical activity).

According to the usual protocol for diagnosing diabetes mellitus, when fasting glucose is higher than 126mg/dL with no unequivocal hyperglycemia, another measurement of fasting glucose should be taken. [15] Since our classification of participants into the diabetic group was based on a one-time measurement at the time of cohort recruitment, there could be limitations in applying this study result to the treatment of patients with diabetes mellitus. [13]

There are two main biological mechanisms related to incidence of colorectal cancer: the hyperinsulinemia hypothesis and the endogenous estradiol-associated hypothesis. Abdominal obesity and type 2 diabetes are associated with metabolic conditions that have been associated with higher risk for cancer and insulin resistance. [20] Obesity is known to be a proinflammatory condition, and inflammation of adipose tissue contributes to increases in proinflammatory cytokine levels and changes in circulating adipokine concentrations. [20] Insulin resistance in adipose tissue, the liver, and skeletal muscle could cause hyperinsulinemia, an increase in insulin production by pancreatic beta cells. [20] Hyperinsulinemia affects the differentiation of intestinal epithelial cells and contributes to colorectal neoplasia. [21] It has been reported in animal studies that the increase of insulin resistance markers such as serum insulin, glucose, fatty acids, and triglycerides could contribute to cancer risk as the energy source and growth factor necessary for the colorectal cancer tissue growth, eventually increasing risk for cancer. [21] C-peptide is produced simultaneously with insulin in the pancreas; its half-life is longer than that of insulin, making it a more stable biomarker. Increases in C-peptide levels have been associated with 37% higher colorectal cancer risk. [22] The overexpression of insulin receptors has been associated with induced colorectal tumors. [23] The expression of estrogen receptors has been closely linked with colorectal cancer progression. [24]

This study has several strengths. First, the order of incidence was made clear by assessing the association of diabetes mellitus and colorectal cancer within a prospective cohort study. Since the median follow-up period for colorectal cancer patients was 11.9 years and the fasting glucose test was done at the time of cohort recruitment, participants were followed for an ample amount of time. Second, we categorized participants as having diabetes mellitus by using both the fasting glucose level measurement from the baseline blood test and self-reported history of diabetes mellitus as criteria for inclusion in the diabetic group, which resulted in higher validity. Thus, we could assess risk for colorectal cancer among diabetes mellitus patients who had not previously been diagnosed. This group of undiagnosed participants turned out to be the largest of all the groups (*n* = 931), demonstrating that control of diabetes mellitus could significantly lower the risk of colorectal cancer. Third, by using data from the baseline survey (at the time of cohort recruitment), there was a lower possibility of confounding due to recall bias. [13] Fourth, the loss-to-follow-up was minimized by linking our data with Korea Central Cancer Registry and death certificate data. [25]

This study has several limitations, which are as follows. First, the statistical power is low. Among the 189 colorectal cancer patients, only 29 had diabetes mellitus (fasting glucose > 126mg/dL), which accounted for 0.2% of study participants. Only 35 participants reported having a previous history of colorectal polyps, and there were no data available to assess the statistical significance of whether the polyp was a precancerous condition. Although information on the anatomical distribution of colorectal cancer was available through the Korea Central Cancer Registry, the number of patients in each sub-site analysis was small, and the statistical power remained low. Second, the assessment of some variables was limited by the information collected by the cohort recruitment questionnaire. For example, no comparison of risk for colorectal cancer associated with type 1 diabetes mellitus versus type 2 diabetes mellitus could be conducted, as the questionnaire did not distinguish between the two types. [13] Nevertheless, from an epidemiologic point of view, the majority of the study population had type 2 diabetes. The prevalence of type 1 diabetes in Korea is 0.017% to 0.021% in the entire population. [26] Additionally, no information on treatment methods, such as use of glucose-lowering medications or insulin injections, was available, and a control rate could not be calculated. However, it was possible to identify how many participants maintained control of their fasting glucose level. Third, the cohort recruitment area included some rural neighborhoods, and 21.0% of study participants were classified as having no formal education. There could have been a differential misclassification bias among participants with a low awareness of diabetes mellitus. However, the possibility of misclassification bias was likely mitigated by classifying participants as having diabetes mellitus according to their fasting glucose level from the blood test. Fourth, the usage of serum glucose and fasting period of at least 6 hours could have influenced the results. It is known that serum glucose value is lower than plasma glucose value by 1.15%. [27] Plasma, rather than serum, is suggested by leading clinical organizations like ADA to measure blood glucose level. Since serum glucose is lower than plasma glucose, the diabetic group being defined as fasting serum glucose ≥ 126mg/dL criteria could have included more participants than measuring plasma glucose level. For this reason, if there is a causal relationship between the high fasting glucose and colorectal cancer, the hazard ratio of colorectal cancer according to the fasting glucose could have been overestimated. In addition, ADA defines fasting as no caloric intake for at least 8 hours. [15] In our study, participants underwent a fasting period of at least 6 hours, which could have led to a higher glucose level. In this case, the effect of hazard ratio of colorectal cancer according to the fasting glucose level is toward the null.

## Conclusion

Both fasting glucose level and self-reported history of diabetes mellitus were associated with increased colorectal cancer risk in the Korean Multi-center Cancer Cohort. In this study, fasting glucose level was more strongly associated with the higher risk for colorectal cancer than self-reported history of diabetes mellitus. This demonstrates that blood test results are supplementary to history of diabetes mellitus, and when both are considered, the result can be explained in a consistent manner in which the risk of colorectal cancer increases.

## Supporting information

S1 Table Hazard ratios (HRs) and 95% confidence intervals (CIs) for colorectal cancer according to fasting glucose level and history of diabetes mellitus (DM) after excluding the first 2 years of follow-up in the Korean Multi-center Cancer Cohort, 1993–2005. (DOCX)

S2 Table Hazard ratios (HRs) and 95% confidence intervals (Cis) for colorectal cancer according to fasting glucose level and history of diabetes mellitus (DM) after excluding the first 2 years of follow-up in the Korean Multi-center Cancer Cohort, 1993–2005. (DOCX)
