# Supplementary material for: Fasting glucose and risk of colorectal cancer in the Korean Multi-center Cancer Cohort
Source: PLoS One. 2017 Nov 21;12(11):e0188465. doi: 10.1371/journal.pone.0188465 (PMC5697863; doi:10.1371/journal.pone.0188465)
Supplement: S1 Table — (DOCX) [file pone.0188465.s001.docx]

**S1 Table.** **Hazard ratios (HRs) and 95% confidence intervals (CIs) for colorectal cancer according to fasting glucose level and history of diabetes mellitus (DM) after excluding the first 2 years of follow-up in the Korean Multi-center Cancer Cohort, 1993-2005**

|  | Both sexes^*^ | | | | Men^**^ | | | | | Women^**^ | | | | | | | |  |
| --- | --- | --- | --- | --- | --- | --- | --- | --- | --- | --- | --- | --- | --- | --- | --- | --- | --- | --- |
|  | Number | CRC cases (n) | Person-years | HR^*^  (95% CI) | Number | CRC cases (n) | Person-years | HR^**^  (95% CI) | Number | | CRC cases (n) | | Person-years | | HR^**^  (95% CI) | |  |  |
| Fasting glucose level | 14,494 | 173 |  |  | 5,828 | 91 |  |  | 8,666 | | 82 | |  | |  | |  |  |
| < 126mg/dL | 13,197 | 145 | 164,393 | 1.00 (Ref.) | 5,283 | 76 | 63,720 | 1.00 (Ref.) | 7,914 | | 69 | | 100,629 | | 1.00 (Ref.) | |  |  |
| ≥ 126mg/dL | 1,297 | 28 | 17,226 | 1.61 (1.07-2.42) | 545 | 15 | 7,114 | 1.58 (0.90-2.76) | 752 | | 13 | | 10,112 | | 1.61 (0.89-2.93) | |  |  |
| Per 10 mg/dL increase |  |  |  | 1.03 (1.00-1.06) |  |  |  | 1.02 (0.97-1.06) |  | |  | |  | | 1.04 (1.00-1.08) | |  |  |
| Self-reported history of diabetes mellitus |  |  |  |  |  |  |  |  |  | |  | |  | |  | |  |  |
| No | 13,770 | 159 | 173,307 | 1.00 (Ref.) | 5,550 | 87 | 67,604 | 1.00 (Ref.) | 8,220 | | 72 | | 105,704 | | 1.00 (Ref.) | |  |  |
| Yes | 724 | 14 | 8,268 | 1.5 (0.87-2.60) | 278 | 4 | 3,231 | 0.77 (0.28-2.10) | 446 | | 10 | | 5,038 | | 2.48 (1.27-4.83) | |  |  |
| Fasting glucose and self-reported history of diabetes mellitus |  | | | |  |  |  |  | |  | |  | |  | |  | | |
| No history of DM &  Glucose < 126mg/dL | 12,845 | 139 | 160,510 | 1.00 (Ref.) | 5,142 | 74 | 62,165 | 1.00 (Ref.) | 7,703 | | 65 | | 98,345 | | 1.00 (Ref.) | |  |  |
| History of DM &  Glucose < 126mg/dL | 352 | 6 | 3,839 | 1.45 (0.64-3.29) | 141 | 2 | 1,555 | 0.85 (0.21-3.48) | 211 | | 4 | | 2,283 | | 2.31 (0.84-6.37) | |  |  |
| History of DM &  Glucose ≥ 126mg/dL | 372 | 8 | 4,430 | 1.69 (0.83-3.45) | 137 | 2 | 1,676 | 0.8 (0.20-3.25) | 235 | | 6 | | 2,754 | | 2.7 (1.17-6.25) | |  |  |
| No history of DM and  Glucose ≥ 126mg/dL | 925 | 20 | 12,797 | 1.61 (1.00-2.58) | 408 | 13 | 5,438 | 1.85 (1.02-3.36) | 517 | | 7 | | 7,358 | | 1.26 (0.57-2.75) | |  |  |

^*^Adjusted for sex and area.

^**^Adjusted for area.
