# Supplementary material for: Fasting glucose and risk of colorectal cancer in the Korean Multi-center Cancer Cohort
Source: PLoS One. 2017 Nov 21;12(11):e0188465. doi: 10.1371/journal.pone.0188465 (PMC5697863; doi:10.1371/journal.pone.0188465)
Supplement: S2 Table — (DOCX) [file pone.0188465.s002.docx]

**S2 Table.** **Hazard ratios (HRs) and 95% confidence intervals (CIs) for colorectal cancer according to fasting glucose level and history of diabetes mellitus (DM) after excluding the first 10 years of follow-up in the Korean Multi-center Cancer Cohort, 1993-2005**

|  | Both sexes^*^ | | | | Men^**^ | | | | | Women^**^ | | | | | | | |  |
| --- | --- | --- | --- | --- | --- | --- | --- | --- | --- | --- | --- | --- | --- | --- | --- | --- | --- | --- |
|  | Number | CRC cases (n) | Person-years | HR^*^  (95% CI) | Number | CRC cases (n) | Person-years | HR^**^  (95% CI) | Number | | CRC cases (n) | | Person-years | | HR^**^  (95% CI) | |  |  |
| Fasting glucose level | 9,936 | 62 |  |  | 3,972 | 30 |  |  | 5,964 | | 32 | |  | |  | |  |  |
| < 126mg/dL | 9,039 | 50 | 128,904 | 1.00 (Ref.) | 3,605 | 24 | 49,887 | 1.00 (Ref.) | 5,434 | | 26 | | 79,017 | | 1.00 (Ref.) | |  |  |
| ≥ 126mg/dL | 897 | 12 | 13,782 | 1.79 (0.95-3.41) | 367 | 6 | 5,616 | 1.57 (0.63-3.91) | 530 | | 6 | | 8,165 | | 1.98 (0.8-4.88) | |  |  |
| Per 10 mg/dL increase |  |  |  | 1.03 (0.98-1.08) |  |  |  | 0.97 (0.87-1.08) |  | |  | |  | | 1.06 (1.00-1.11) | |  |  |
| Self-reported history of diabetes mellitus |  |  |  |  |  |  |  |  |  | |  | |  | |  | |  |  |
| No | 9,577 | 59 | 137,498 | 1.00 (Ref.) | 3,827 | 30 | 53,367 |  | 5,750 | | 29 | | 84,131 | | 1.00 (Ref.) | |  |  |
| Yes | 359 | 3 | 5,187 | 1.15 (0.36-3.69) | 145 | 0 | 2,136 |  | 214 | | 3 | | 3,051 | | 2.8 (0.85-9.27) | |  |  |
| Fasting glucose and self-reported history of diabetes mellitus |  | | | |  |  |  |  | |  | |  | |  | |  | | |
| No history of DM &  Glucose < 126mg/dL | 8,881 | 49 | 126,681 | 1.00 (Ref.) | 3,537 | 24 | 48,916 | 1.00 (Ref.) | 5,344 | | 25 | | 77,766 | | 1.00 (Ref.) | |  |  |
| History of DM &  Glucose < 126mg/dL | 158 | 1 | 2,223 | 1.07 (0.15-7.74) | 68 | 0 | 971 |  | 90 | | 1 | | 1,251 | | 2.73 (0.37-20.3) | |  |  |
| History of DM &  Glucose ≥ 126mg/dL | 201 | 2 | 2,964 | 1.39 (0.34-5.76) | 77 | 0 | 1,165 |  | 124 | | 2 | | 1,800 | | 3.13 (0.74-13.3) | |  |  |
| No history of DM and  Glucose ≥ 126mg/dL | 696 | 10 | 10,817 | 1.91 (0.96-3.82) | 290 | 6 | 4,452 | 1.95 (0.78-4.89) | 406 | | 4 | | 6,366 | | 1.72 (0.59-5.02) | |  |  |

^*^Adjusted for sex and area.

^**^Adjusted for area.
